# Nitration of ovalbumin decreases the risk for sensitization via the oral route in a mouse food allergy model

**DOI:** 10.1186/2045-7022-1-S1-O49

**Published:** 2011-08-12

**Authors:** Susanne C Diesner, Eva Untersmayr, Gertie J Oostingh, Kathrin Selzle, Tobias Pfaller, Cornelia Schultz, Yingyi Zhang, Durga Krishnamurthy, Philipp Starkl, Regina Knittelfelder, Elisabeth Förster-Waldl, Arnold Pollak, Otto Scheiner, Ulrich Pöschl, Erika Jensen-Jarolim, Albert Duschl

**Affiliations:** 1 Medical University of Vienna, Department of Pathophysiology and Allergy Research and Department of Pediatrics, Vienna, Austria; 2 Medical University of Vienna, Department of Pathophysiology and Allergy Research, Vienna, Austria; 3 University of Salzburg, Department of Molecular Biology, Salzburg, Austria; 4 Max Planck Institute for Chemistry, Biogeochemistry Department, Mainz, Germany; 5 Medical University of Vienna, Department of Pediatrics and Adolescent Medicine, Vienna, Austria

## Background

Previously, nitration e.g. by ambient pollutants was demonstrated to increase the allergenicity of the major birch pollen allergen Bet v1. As also endogenous nitration during inflammation could influence food protein immunogenicity and contribute to food allergic reactions, we aimed to analyze the impact of protein nitration on sensitization in a murine food allergy model.

## Methods and results

BALB/c mice were fed untreated (OVA), sham-nitrated (snOVA) or nitrated ovalbumin (nOVA) with or without concomitant acid-suppression. To analyze systemic effects, mice were injected the allergens intraperitoneally (i.p.). Animals being fed OVA or snOVA with antiacids developed elevated IgE, IgG1 and IgG2a titers. Oral immunizations of nOVA under acid-suppression did not result in IgG and IgE formation. However, all i.p. immunized mice revealed high levels of IgE, which were significantly increased in the group being injected nOVA. In RBL-assays, all groups with OVA-specific IgE showed a significant increased mediator release with nOVA as trigger compared to OVA. To analyze the immune response in the involved organ, gastric tissues were screened for cytokine expression by real-time-PCR. Only the acid-suppressed groups being fed OVA or snOVA revealed a higher expression of Th2 and inflammatory markers. In gastric digestion experiments, nOVA was degraded within minutes, whereas OVA and snOVA remained stable up to 120min. Additionally, HPLC-chip-MS/MS analysis revealed the most efficiently nitrated tyrosine residue within an ovalbumin epitope recognized exclusively after oral immunization.

## Conclusion

Despite its enhanced allergenicity nOVA has a reduced oral sensitization potential due to enhanced protein digestibility and/or changes in antibody epitopes.

